# Second-Line Systemic Treatment for Metastatic Urothelial Carcinoma: A Network Meta-Analysis of Randomized Phase **III** Clinical Trials

**DOI:** 10.3389/fonc.2019.00679

**Published:** 2019-07-25

**Authors:** Hyun Sik Yoon, Cheol Kwak, Hyeon Hoe Kim, Hyung Suk Kim, Ja Hyeon Ku

**Affiliations:** ^1^Department of Urology, Dongguk University College of Medicine, Goyang, South Korea; ^2^Department of Urology, Seoul National University College of Medicine, Seoul, South Korea

**Keywords:** carcinoma, transitional cell, chemotherapy, immunotherapy, survival, network meta-analysis

## Abstract

**Purpose:** We aimed to evaluate and compare relative impacts of various second-line treatments on overall survival (OS) in metastatic urothelial carcinoma (mUC).

**Method:** A literature search was conducted in PubMed, Embase, and the Cochrane Library for all articles published prior to December 2018 in accordance with the Preferred Reporting Items for Systematic Review and Meta-analysis guidelines. Seven randomized controlled trials with phase III design that met study eligibility criteria were selected for final analysis. A Bayesian framework network meta-analysis (NMA) was applied to indirectly compare the effect of each treatment on OS.

**Results:** In NMA, atezolizumab (HR, 0.90; 95% CI, 0.57–1.40) and pembrolizumab (HR, 0.77, 95% CI, 0.48–1.20) showed no significant effect on OS improvement compared to vinflunine. Gemcitabine/paclitaxel combination (HR, 1.30; 95% CI, 0.80–1.90) and lapatinib (HR, 0.95; 95% CI, 0.57–1.60) was not significantly associated with OS improvement compared to atezolizumab and best supportive care, respectively. However, results of rankograms revealed that pembrolizumab and atezolizumab were the first and second rank therapeutic agents for OS improvement in post-platinum mUC.

**Conclusions:** Our NMA results are inconclusive. The optimal second-line treatment for OS improvement could not be determined because there were no significant OS differences among evaluated therapeutic agents. However, the use of immunotherapeutic agents such as atezolizumab and pembolizumab may have priority for improving OS in second-line setting of mUC.

## Introduction

Urothelial carcinoma (UC) originating from the bladder or upper urinary tracts (renal pelvis or ureter) is the most common histologic type of cancer. It generally shows chemo-sensitive feature. On the basis of these chemo-sensitivity of UC, platinum (cisplatin or carboplatin) based combination chemotherapy has long been used as first-line standard treatment for metastatic UC (mUC) (1, 2). With cisplatin-based regimens, 40–60% overall response rate (ORR) and median overall survival (OS) of 14–15 months can be expected for mUC patients (3–5). For mUC patients who are unfit for cisplatin-based regimens because of multiple comorbidities (i.e., neuropathy, hearing loss), poor performance status, or impaired renal function, carboplatin-based regimens are primarily applied as a feasible option, showing an 30–40% ORR and median OS of 9–10 months that are inferior results than those of cisplatin-based regimens (6, 7).

In spite of these efficacies of first-line treatments for mUC patients, a considerable number of patients experience disease progression during or after fist-line treatments. Therefore, they will require second-line therapy. Although several chemotherapeutic agents have been investigated in the second-line setting of mUC, they have only presented marginal activity with ORR of <20% and median OS of <9 months with considerable toxicity profiles (8, 9). Furthermore, there has been no evidence that second-line chemotherapy may improve OS or quality of life (10). Consequently, currently there are no approved second-line chemotherapeutic agents for mUC in the United States. According to current NCCN guidelines, paclitaxel and/or gemcitabine is the recommended second-line chemotherapeutic agents in post-platinum mUC due to higher ORR (~40%) observed in a previous phase III study (11, 12). Vinflunine, a novel vinca alkaloid, is the only approved chemotherapeutic agent in the European Union based on results of phase III trials performed in the second-line setting of mUC (13–15).

In the last decades, with an increasing understanding of molecular biology and cancer immunobiology, research on systemic therapy in the oncologic field has mainly focused on targeted and immunotherapeutic agents other than cytotoxic chemotherapy (9). Lapatinib, a dual tyrosine kinase inhibitor (TKI) that targets human epidermal growth factor receptor (HER) pathway, has been evaluated as a possible second-line therapy in mUC (16). Besides, several immunotherapeutic agents that can block immune checkpoints, such as programmed cell death 1 (PD-1) or PD-ligand-1 (PD-L1), have been investigated in the second-line setting of mUC (17). Among these, atezolizumab (PD-L1 inhibitor) and pembrolizumab (PD-1 inhibitor) were approved by US-Food and Drug Administration (US-FDA) as the first or second line treatment in mUC based on durable therapeutic response and tolerable safety profiles observed in previous clinical trials (18, 19). However, consensus has not been reached yet regarding which second-line agent is the optimal treatment in terms of survival benefit in mUC.

Thus, the objective of the present study was to assess and compare the efficacy of each second-line treatment on OS improvement for determining the optimal therapeutic agent in post-platinum mUC setting. To achieve this goal, we conducted a network meta-analysis (NMA) of available data by only including phase III, randomized clinical trials (RCTs).

## Materials and Methods

The present NMA was performed and reported in line with recommendations of the preferred reporting items for systematic reviews and meta-analyses (PRISMA) statement (20).

### Search Strategy

We conducted an electronic search for clinical trials on second-line systemic therapeutic agents in mUC prior to December 2018 using PubMed, Embase, and the Cochrane Library. The search was limited to English articles with full-text publications. Search terms were used separately or in combination as followings: *(metastatic bladder cancer OR metastatic urothelial carcinoma OR metastatic bladder carcinoma) AND (systemic chemotherapy OR systemic treatment OR immune checkpoint inhibitor)*. Citation lists of all searched articles were then used to confirm other possible relevant publications. Only studies with well-established study design with comparative arms and explicit description of patients' characteristics were finally selected. Two independent reviewers (HSK and CK) selected these articles. Any disagreements among reviewers were settled by consensus with a third reviewer (HHK).

### Eligible Criteria

Study eligibility was defined according to Population, Intervention, Comparator, Outcome, and Study design (PICOS) system (20): *Population*, Patients with mUC; *Interventions*, Second-line treatment after first-line chemotherapy; *Comparators*, Another second-line treatment (i.e., placebo, best supportive care); *Outcome*, OS; *Study design*, prospective RCTs with phase III design.

Articles were eligible if they met following inclusion criteria: (1) human research; (2) patients with mUC previously treated with the first-line systemic chemotherapy; (3) received second-line systemic treatment; (4) reported outcome value (OS); (5) available assessment for the association between second-line treatment and OS; (6) sufficient information provided to estimate hazard ratio (HR) and their 95% confidence interval (CI); and (7) RCTs. Exclusion criteria were as followings: review articles, letters, editorial comments, case reports, and articles that did not provide raw data.

### Data Extraction

Three independent reviewers (HSY, HSK, and JHK) extracted the required information from all eligible studies and then compared their results to confirm accuracy. Any disagreements for extracted data between two reviewers were settled by consensus. Extracted data were recorded in accordance with reporting recommendations for tumor marker prognostic studies (REMARK) guidelines (21) as follows: (1) publication data including the name of the first author, year of publication, country, and recruitment period; (2) study characteristics including the number of patients, median age, and gender distribution in treatment and control groups, study endpoints, and duration of median follow-up; and (3) treatment characteristics including regimens, dosage of regimens, number of planned cycles, median OS, HR for OS with 95% CI, and percentage of grade 3 to grade 4 toxicity.

### Statistical Analysis

To indirectly compare the effect of each second-line therapeutic agent on OS, we performed NMA using a Bayesian model and Markov chain Monte Carlo methods called Gibbs sampling conducted in WinBUGS 1.4 (MRC Biostatistics Unit, Cambridge, UK) (22). Either a fixed or random effects model for reported outcomes was selected according to model fit criteria (Deviance Information Criteria, DIC) for penalizing greater model complexity (23). We modeled binary variables for every treatment group of every study. Results of NMA on OS were specified as HRs with 95% credible intervals (CrIs) across studies. Each analysis was based on non-informative priors for effect size and precision. We also suggested surface under the cumulative ranking (SUCRA) that represented ranking probabilities to provide a hierarchy of treatments accounting for both the location and the variance of all relative treatment effects (24), with higher value indicating better treatment ranking. Publication bias was explored using the funnel plot, the Egger's and the Begg's test. A symmetrical inverted funnel indicates no significant publication bias. Whereas, in the presence of publication bias, inverted funnel shows skewed and asymmetrical pattern. Besides, publication bias is significantly suspected if the *p*-values for the Begg's and Egger's tests are <0.05 (25, 26). The Bayesian framework NMA was implemented with NetMetaXL which provided an interface using WinBUGS within Microsoft Excel (27). All *p*-values were two-sided and *p* < 0.05 was considered statistically significant.

## Results

### Literature Search Results

**Figure 1:**
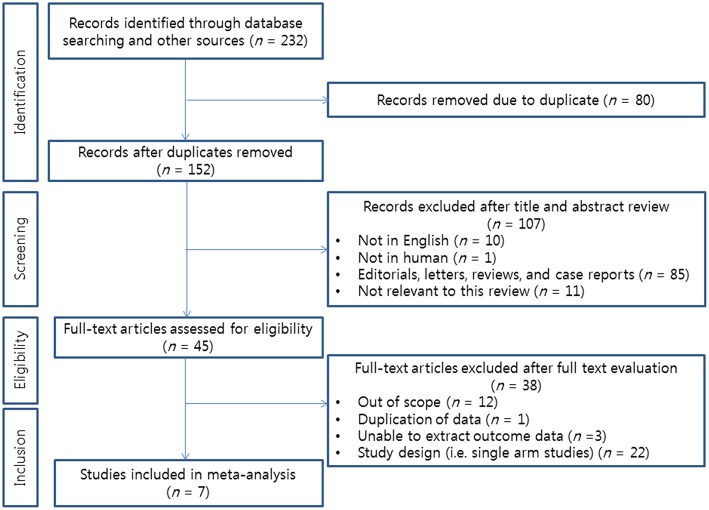
PRISMA flow diagram describing the search strategy used for network meta-analysis.

We identified 232 articles after initial database searches. Among these, 80 duplicated publications were excluded. After reviewing titles and abstracts, 107 articles were also excluded. Thus, a total of 45 articles remained for full text review. According to inclusion criteria of our analysis, a total of 7 RCTs were finally selected for the current NMA (12–16, 18, 19). The PRISMA flow diagram depicting the process for literature search and selection of studies is presented in Figure 1.

### Overview of Included Studies

#### Study Characteristics

Characteristics of each included study are summarized in Table 1. All studies were phase III prospective RCTs published between 2009 and 2018. The recruitment period of patients ranged from 2001 to 2016. Randomization of patients to the treatment group and control group was made at ratio of 1:1 (12, 16, 18, 19) or 2:1 (13–15). Most studies considered OS as primary endpoint except for one study (16). Among these 7 studies, three studies (13–15) consisted of the same mUC cohort using specific agent (vinflunine) as second-line treatment. One was an original study (13). Another study presented long-term survival results with extended follow-up duration for the original study (14). The remaining study reported results of subgroup analysis conducted for mUC patients treated with prior cisplatin (15). Further characteristics of these eligible studies can be identified in Table 1.

**Table 1:** Study characteristics of the eligible phase III randomized controlled trials for network meta-analysis.

**Study**	**Year**	**Country**	**Recruitment period**	**Total patients (ITT)**	**Median age, years (range)**	**No. of gender (male/female)**	**Endpoints (primary/secondary)**	**Median follow-up duration (months)**
Albers et al. (12)	2011	Germany	2001–2005	Treatment arm: 48 Control arm: 48	Treatment arm: 63.9 (42.8–80.6) Control arm: 65.1 (42.8–79.4)	NA	OS/PFS, ORR, toxicity	NA
Bellmunt et al. (13) (NCT00315237)	2009	Europe and USA	2003–2006	Treatment arm: 253 Control arm: 117	64.3 (34.9–86.3)	NA	OS/ORR, PFS, DCR	Treatment arm: 21.5 Control arm: 22.3
Bellmunt et al. (14)	2013	Europe and USA	2003–2006	Treatment arm: 253 Control arm: 117	64.3 (34.9–86.3)	NA	OS/NA	Treatment arm: 42 Control arm: 45
Harshman et al. (15)	2013	Europe and USA	2003–2006	Treatment arm: 167 Control arm: 84	62.5 (34.6–82.3)	NA	OS/NA	Treatment arm: 21.5 Control arm: 22.3
Powles et al. (16) (NCT00949455)	2017	United Kingdom	2007–2013	Treatment arm: 116 Control arm: 116	Treatment arm: 70.7 (63.9–77.2) Control arm: 71.1 (63.8–76.3)	Treatment arm: 88/28 Control arm: 84/32	PFS/OS, ORR, toxicity	NA
Bellmunt et al. (18) (NCT02256436, KEYNOTE-045)	2017	Multi-nation	2014–2015	Treatment arm: 270 Control arm: 272	Treatment arm: 67 (29–88) Control arm: 65 (26–84)	Treatment arm: 200/70 Control arm: 202/70	OS, PFS/ORR, DOR, toxicity	14.1
Powles et al. (19) (NCT02302807, IMvigor211)	2018	Multi-nation	2015–2016	Treatment arm: 467 Control arm: 464	Treatment arm: 67 (33–88) Control arm: 67 (31–84)	Treatment arm: 357/110 Control arm: 361/103	OS/PFS, ORR, DOR, toxicity	17.3

*OS, overall survival; ORR, overall response rate; PFS, progression-free survival; DCR, disease control rate; NA, non-available, DOR; duration of response*.

### Treatment Characteristics

Details on treatment characteristics of these eligible 7 studies are shown in Table 2. Second-line agents evaluated in treatment arms were as followings: vinflunine (13–15), gemcitabine/paclitaxel (GP) (12), lapatinib (16), pembrolizumab (18), and atezolizumab (19). The number of cycles was not clearly mentioned in most of these studies. Generally, the median OS ranged from 6.9 to 12.6 months in treatment arms and from 4.3 to 12.0 months in control arms. Among these assessed second-line agents, only two drugs (vinflunine, pembrolizumab) showed significant OS benefit relative to each control group (best supportive care, chemotherapeutic agents) (13, 18). The use of prolonged GP was significantly associated with higher treatment-related toxicity compared to short-term GP (12). In contrast, immune checkpoint inhibitors (ICIs) including pembrolizumab and atezolizumb presented lower toxicity profiles than second-line chemotherapy (18, 19).

**Table 2:** Treatment characteristics of the eligible phase III randomized controlled trials for network meta-analysis.

**Study**	**Treatment arm**	**Control arm**	**Dose of regimens (mg/m^**2**^)**	**No. of planned cycles**	**Median OS, months (treatment/control) (*p*-value)**	**HR for OS (95% CI)**	**Grade 3–4 Toxicity, % (treatment/control)**
Albers et al. (12)	Short-term GP	Prolonged GP	Gemcitabine: 1,000 Paclitaxel: 175	6	7.8/8.0 (0.772)	NA	6.6/26.6
Bellmunt et al. (13) (NCT00315237)	Vinflunine	BSC alone	320 or 280	NA	6.9/4.3 (0.040)	0.77 (0.61–0.98)	19.3/17.9
Bellmunt et al. (14)	Vinflunine	BSC alone	320 or 280	NA	6.9/4.3 (0.023)	0.78 (0.61–0.96)	NA
Harshman et al. (15)	Vinflunine	BSC alone	320 or 280	NA	6.9/4.7 (0.043)	0.76 (0.58–0.99)	NA
Powles et al. (16) (NCT00949455)	Lapatinib	Placebo	1,500 (fixed dose)	NA	12.6/12.0 (0.80)	0.96 (0.70–1.31)	8.6/8.1
Bellmunt et al. (18) (NCT02256436, KEYNOTE-045)	Pembrolizumab	Investigator's choice of chemotherapy (paclitaxel, docetaxel, or vinflunine)	Pembrolizumab: 200 (fixed dose) Paclitaxel: 175 Docetaxel: 75 Vinflunine: 320	NA	10.4/7.4 (0.002)	0.73 (0.59–0.91)	15.0/49.4
Powles et al. (19) (NCT02302807, IMvigor211)	Atezolizumab	Investigator's choice of chemotherapy (paclitaxel, docetaxel, or vinflunine)	Atezolizumab: 1,200 (fixed dose) Paclitaxel: 175 Docetaxel: 75 Vinflunine: 320	NA	11.1/10.6 (0.41)	0.87 (0.63–1.21)	20/43

*BSC, best supportive care; GP, gemcitabine and paclitaxel; NA, non-available; OS, overall survival; HR, hazard ratio; CI, confidence interval*.

### Bayesian Framework Network Meta-Analysis

**Figure 2:**
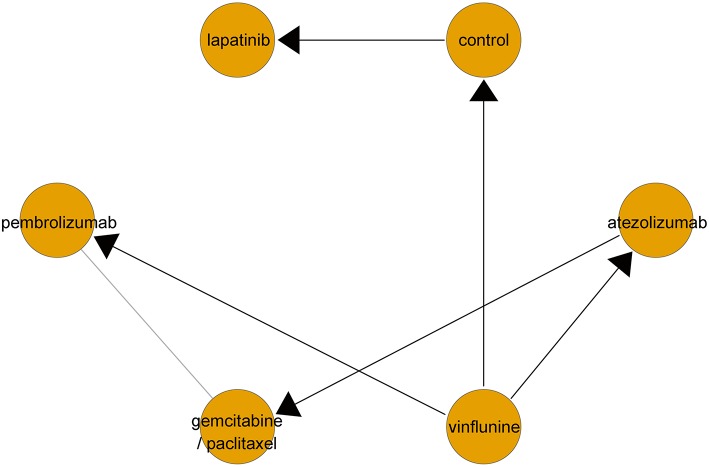
Network geometry of clinical trials on second-line therapeutic agents for overall survival in metastatic urothelial carcinoma. Lines with arrows represent direct comparison between two treatments (set the side from which the arrow leaves as control arm). Gray line implies indirect comparison between two treatments.

Networks for indirect comparisons among multiple second-line treatments in terms of OS are described in Figure 2. A fixed effects model was applied since the DIC of the fixed effects model was lower than that of the random effects model.

**Figure 3:**
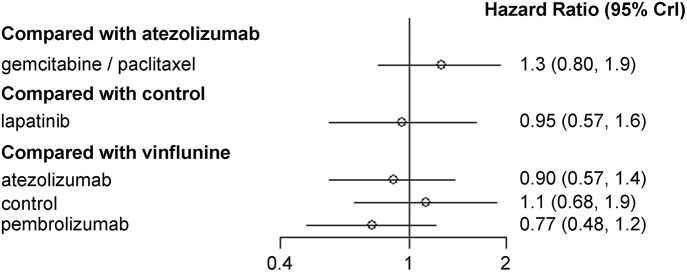
Pooled hazard ratio and 95% credible intervals for network meta-analysis of the impact of each second-line therapeutic agent on overall survival.

Results of NMA are depicted in Figure 3. When vinflunine was used as the reference for comparison, atezolizumab (HR, 0.90; 95% CrI, 0.57–1.40) and pembrolizumab (HR, 0.77, 95% CrI, 0.48–1.20) showed no significant efficacy in terms of OS benefit. Likewise, GP combination had no significant effect on OS (HR, 1.30; 95% CrI, 0.80–1.90) compared with atezolizumab. There was no significant difference in OS between lapatinib and control (best supportive care; BSC) either (HR, 0.95; 95% CrI, 0.57–1.60).

**Figure 4:**
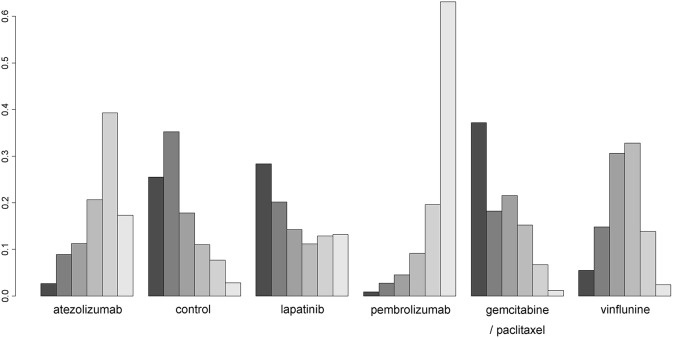
Rankograms for second-line treatment network of overall survival. The size of each bar corresponds to the probability of each treatment to be at a specific rank.

Figure 4 presents SUCRA plots of six different second-line treatments (including control) in terms of OS benefit. Despite the lack of statistical significance in results of NMA, SUCRA analyses revealed that pembrolizumab and atezolizumab had high likelihood of being ranked first (~65% probability) and second (about 40% probability), respectively. However, GP combination and lapatinib were most likely to be ranked the worst, inferior to the control.

### Publication Bias

There was no strong evidence of publication bias in that the funnel plot showed a certain degree of symmetry in the NMA of OS (Supplementary Figure 1). Also, the Begg's and Egger's tests demonstrated that there was no statistically significant publication bias in the NMA of OS (all *p*-values > 0.05; Supplementary Figure 1).

## Discussion

Although platinum (cisplatin or carboplatin)-based combination chemotherapy has been used as the primary treatment to improve survival outcomes in mUC patients, a substantial number of patients have experienced disease progression during or after the first-line platinum-based combination chemotherapy. Thus, secondary treatment is usually considered for such patients (1, 2). Several single chemotherapeutic agents have been investigated in second-line setting of mUC. However, no survival benefit, poor response rate, and significant treatment related-toxicity have been reported in association with second-line chemotherapy (8, 9). Currently, there are no US-FDA approved second-line chemotherapeutic agents for mUC.

Several therapeutic agents have been explored in phase III RCT design at second-line setting of mUC. Bellmunt et al. (13, 14) have reported that vinflunine with best supportive care (BSC) show a modest ORR of 8.6% and a meaningful survival gain of 2.6 months compared with BSC alone. Based on these results, vinflunine is currently the only second-line chemotherapeutic agent in mUC approved by European Medicines Agency in Europe. Short-term vs. prolonged second-line treatment using GP combination has been compared in a phase III RCT (12). Results showed that OS (7.8 vs. 8.0 months) was similar in both groups. However, treatment-related toxicity rate was relatively higher in the prolonged GP group (26.6%) than that in the short-term GP group (6.6%) (12). Irrespective of treatment duration, high response rate of up to 40% was observed in each group (37.5% in short-term GP vs. 41.5% in prolonged GP) (12). There have been studies on other agents as second-line treatment besides cytotoxic chemotherapy drugs. Lapatinib known as a TKI of HER pathway has been compared with placebo in second-line setting of mUC. Results of that study demonstrated that no significant differences in end-points of interest such as median progression-free survival (4.5 vs. 5.1 months), OS (12.6 vs. 12.0 months), or rate of grade 3–4 toxicities (8.6 vs. 8.1%) between the two groups (16). In recent years, systemic immunotherapy represented by immune checkpoint inhibition (ICI) has been vigorously assessed as a promising therapeutic option for various metastatic solid tumors, including mUC. Several comparative studies of ICI drugs and cytotoxic chemotherapy as second-line treatment in mUC through phase III RCT have been reported (18, 19). In KEYNOTE-045 trial, pembrolizumab (PD-1 inhibitor) showed superior response rate (21.1 vs. 11.4%), significant OS benefit (10.3 vs. 7.4 months), and better tolerable grade 3 or more toxicity profiles (15.0 vs. 49.4%) compared to chemotherapy (18). However, atezolizumab (PD-L1 inhibitor) did not show significant difference in terms of OS (11.1 vs. 10.6 months) or response rate (13.4% in each group) relative to chemotherapy in IMvigor 211 trial, although safety profiles (grade 3–4 toxicities) of atezolizumab were more favorable than chemotherapy (20 vs. 43%) (19). Notably, commonly observed feature in ICI related studies is that the response rate to treatment is not high (ranged from 10 to 20%). However, if response is observed, the response tends to be maintained during the treatment period (17).

However, there is no consensus on which of these drugs is the optimal second-line treatment for mUC. In the current study, we sought to evaluate the optimal second-line therapeutic agent in terms of OS improvement by indirectly comparing agents mentioned above. To the best of our knowledge, this is the first report pooling multiple second-line treatment agents of mUC (vinflunine, GP combination, lapatinib, atezolizumab, and pembrolizumab) assessed in previous phase III RCTs (12–16, 18, 19). NMA was applied for indirect comparison among these included drugs and SUCRA analysis was used to rank these included agents. Our NMA results showed no statistically significant OS differences among these assessed agents. However, SUCRA analysis demonstrates that it is highly likely that ICI drugs, including pembrolizumab and atezolizumab, are the preferred second-line therapeutics in mUC. A recently published study (26) using NMA has pooled previous three RCTs (13, 18, 19) and reported that all three drugs (vinflunine, pembrolizumab, and atezolizumab) showed a significant response rate compared with BSC. However, the rate of treatment discontinuation due to side effect was significantly higher in chemotherapy than that in BSC. In comparison with BSC, two ICI drugs demonstrated no significant treatment stop rate for adverse events while only pembrolizumab was associated with significant OS benefit (28). These analytic results suggest that ICI has durable therapeutic response and tolerable safety profiles. Thus, ICI drugs may be considered as the first option for second-line treatment of mUC (11, 17). Currently, there are several ongoing clinical trials which evaluate the combined treatment strategies with ICI and other therapeutic modalities, such as chemotherapy, target therapy, and other ICI (i.e., anti-CTLA-4 inhibitor), in the first or second-line and beyond setting of mUC (Supplementary Table 1). There have been also several clinical trials evaluating the inhibition of growth factor receptor (GFR) as a promising therapeutic target in mUC (29, 30) (Supplementary Table 1). Ramucirumab, which is a human IgG1 antibody to vascular-endothelial GFR-2 (VEGFR-2), and docetaxel combination presented favorable progression-free survival than chemotherapy plus placebo in patients with platinum-refractory mUC as a result of phase III RCT (29). Recently, erdafitinib, a pan-fibroblast GFR (FGFR) inhibitor, was granted accelerated US-FDA approval for the use in patients with locally advanced or metastatic UC with susceptible FGFR3 or FGFR2 genetic alterations who have progressed during or after platinum-containing chemotherapy based on the results of a phase II clinical trial (30). When the results of these ongoing clinical trials are published, it is expected to provide evidence for novel treatment strategies for mUC, further changing the treatment paradigm.

The present study has some limitations. First, although our study only included previously reported phase III RCTs for NMA, the association between second-line treatments and OS could not be controlled through multivariate analysis with other variables such as treatment-induced toxicity and patient dropout which might have affected results of this study. Besides, there might be differences in patient background among anticancer agents. Although these differences could not be easily identified and adjusted, they might have effect on the results of the study if considered when agents are compared. Second, the primary end-point of this analysis only included OS improvement. Therefore, we did not assess the relationship between second-line therapeutic agents and other clinical outcomes, including treatment-related side effects and response rates. Even if the final goal of cancer treatment is to improve the survival of patients, adverse events, and response rates for treatment might have significant impact on the choice of treatment modality. If these factors were considered as other end-points in the analysis, it might be possible to provide a more crucial basis for finding the optimal second-line therapeutic agent in mUC. Lastly, the current study only included full-text articles published in English which might lead to language bias (31), although there was no evidence of publication bias in the present study.

## Conclusions

Results of our study were inconclusive in view of the inability to determine the optimal second-line treatment for OS improvement because there were no significant OS differences among evaluated therapeutic agents based on results of NMA. However, the use of ICI drugs such as atezolizumab and pembolizumab may be given priority for improving OS in second-line setting of mUC.

## Data Availability

All datasets generated for this study are included in the manuscript and/or the Supplementary Files.

## Author Contributions

HSK, JK, CK, and HHK: conception and design and supervision. HY, HSK, and JK: data acquisition, manuscript drafting, and statistical analysis. JK and HSK: data analysis and interpretation and critical revision of the manuscript for scientific and factual content.

### Conflict of Interest Statement

The authors declare that the research was conducted in the absence of any commercial or financial relationships that could be construed as a potential conflict of interest.

## Supplementary Material

Supplementary Table 1 Ongoing clinical trials for immune checkpoint inhibitors in metastatic UC^a^.

Supplementary Figure 1 Funnel plot for the assessment of potential publication bias in enrolled studies. Each point means a separate study for the indicated association. Vertical line indicates the mean effects size.

## References

[1] Alfred Witjes J Lebret T Comperat EM Cowan NC De Santis M Bruins HM. Updated 2016 EAU guidelines on muscle-invasive and metastatic bladder cancer. Eur Urol. (2017) 71:462–75. 10.1016/j.eururo.2016.06.020 27375033

[2] Bellmunt J Orsola A Leow JJ Wiegel T De Santis M Horwich A. Bladder cancer: ESMO practice guidelines for diagnosis, treatment and follow-up. Ann Oncol. (2014) 25:iii40–8. 10.1093/annonc/mdu223 25096609

[3] Logothetis CJ Dexeus FH Finn L Sella A Amato RJ Ayala AG. A prospective randomized trial comparing MVAC and CISCA chemotherapy for patients with metastatic urothelial tumors. J Clin Oncol. (1990) 8:1050–5. 10.1200/JCO.1990.8.6.1050 2189954

[4] Loehrer PJ Sr Einhorn LH Elson PJ Crawford ED Kuebler P Tannock I A randomized comparison of cisplatin alone or in combination with methotrexate, vinblastine, and doxorubicin in patients with metastatic urothelial carcinoma: a cooperative group study. J Clin Oncol. (1992) 10:1066–73. 10.1200/JCO.1992.10.7.1066 1607913

[5] von der Maase H Sengelov L Roberts JT Ricci S Dogliotti L Oliver T. Long-term survival results of a randomized trial comparing gemcitabine plus cisplatin, with methotrexate, vinblastine, doxorubicin, plus cisplatin in patients with bladder cancer. J Clin Oncol. (2005) 23:4602–8. 10.1200/JCO.2005.07.757 16034041

[6] Dogliotti L Carteni G Siena S Bertetto O Martoni A Bono A. Gemcitabine plus cisplatin versus gemcitabine plus carboplatin as first-line chemotherapy in advanced transitional cell carcinoma of the urothelium: results of a randomized phase 2 trial. Eur Urol. (2007) 52:134–41. 10.1016/j.eururo.2006.12.029 17207911

[7] De Santis M Bellmunt J Mead G Kerst JM Leahy M Maroto P. Randomized phase II/III trial assessing gemcitabine/carboplatin and methotrexate/carboplatin/vinblastine in patients with advanced urothelial cancer who are unfit for cisplatin-based chemotherapy. J Clin Oncol. (2012) 30:191–9. 10.1200/JCO.2011.37.3571 22162575 PMC3255563

[8] Sonpavde G Sternberg CN Rosenberg JE Hahn NM Galsky MD Vogelzang NJ. Second-line systemic therapy and emerging drugs for metastatic transitional-cell carcinoma of the urothelium. Lancet Oncol. (2010) 11:861–70. 10.1016/S1470-2045(10)70086-3 20537950

[9] Oing C Rink M Oechsle K Seidel C von Amsberg G Bokemeyer C. Second line chemotherapy for advanced and metastatic urothelial carcinoma: vinflunine and beyond—a comprehensive review of the current literature. J Urol. (2016) 195:254–63. 10.1016/j.juro.2015.06.115 26410730

[10] Dreicer R. Second-line chemotherapy for advanced urothelial cancer: because we should or because we can? J Clin Oncol. (2009) 27:4444–5. 10.1200/JCO.2009.23.8071 19687324

[11] Spiess PE Agarwal N Bangs R Boorjian SA Buyyounouski MK Clark PE. Bladder cancer, version 5.2017, NCCN clinical practice guidelines in oncology. J Natl Compr Canc Netw. (2017) 15:1240–67. 10.6004/jnccn.2017.0156 28982750

[12] Albers P Park SI Niegisch G Fechner G Steiner U Lehmann J. Randomized phase III trial of 2nd line gemcitabine and paclitaxel chemotherapy in patients with advanced bladder cancer: short-term versus prolonged treatment [German Association of Urological Oncology (AUO) trial AB 20/99]. Ann Oncol. (2011) 22:288–94. 10.1093/annonc/mdq398 20682548

[13] Bellmunt J Theodore C Demkov T Komyakov B Sengelov L Daugaard G. Phase III trial of vinflunine plus best supportive care compared with best supportive care alone after a platinum-containing regimen in patients with advanced transitional cell carcinoma of the urothelial tract. J Clin Oncol. (2009) 27:4454–61. 10.1200/JCO.2008.20.5534 19687335

[14] Bellmunt J Fougeray R Rosenberg JE von der Maase H Schutz FA Salhi Y. Long-term survival results of a randomized phase III trial of vinflunine plus best supportive care versus best supportive care alone in advanced urothelial carcinoma patients after failure of platinum-based chemotherapy. Ann Oncol. (2013) 24:1466–72. 10.1093/annonc/mdt007 23419284

[15] Harshman LC Fougeray R Choueiri TK Schutz FA Salhi Y Rosenberg JE. The impact of prior platinum therapy on survival in patients with metastatic urothelial cancer receiving vinflunine. Br J Cancer. (2013) 109:2548–53. 10.1038/bjc.2013.617 24129239 PMC3833211

[16] Powles T Huddart RA Elliott T Sarker SJ Ackerman C Jones R. Phase III, double-blind, randomized trial that compared maintenance lapatinib versus placebo after first-line chemotherapy in patients with human epidermal growth factor receptor 1/2-positive metastatic bladder cancer. J Clin Oncol. (2017) 35:48–55. 10.1200/JCO.2015.66.3468 28034079

[17] Kim HS Seo HK. Immune checkpoint inhibitors for urothelial carcinoma. Investig Clin Urol. (2018) 59:285–96. 10.4111/icu.2018.59.5.285 30182073 PMC6121021

[18] Bellmunt J de Wit R Vaughn DJ Fradet Y Lee JL Fong L. Pembrolizumab as second-line therapy for advanced urothelial carcinoma. N Eng J Med. (2017) 376:1015–26. 10.1056/NEJMoa1613683 28212060 PMC5635424

[19] Powles T Durán I van der Heijden MS Loriot Y Vogelzang NJ De Giorgi U. Atezolizumab versus chemotherapy in patients with platinum-treated locally advanced or metastatic urothelial carcinoma (IMvigor211): a multicentre, open-label, phase 3 randomised controlled trial. Lancet. (2018) 391:748–57. 10.1016/S0140-6736(17)33297-X 29268948

[20] Moher D Liberati A Tetzlaff J Altman DG. Preferred reporting items for systematic reviews and meta-analyses: the PRISMA statement. Ann Intern Med. (2009) 151:264–9. 10.7326/0003-4819-151-4-200908180-00135 19622511

[21] Altman DG McShane LM Sauerbrei W Taube SE. Reporting recommendations for tumor marker prognostic studies (REMARK): explanation and elaboration. BMC Med. (2012) 10:51. 10.1186/1741-7015-10-51 22642691 PMC3362748

[22] Lu G Ades AE. Combination of direct and indirect evidence in mixed treatment comparisons. Stat Med. (2004) 23:3105–24. 10.1002/sim.1875 15449338

[23] Caldwell DM Ades AE Higgins JP. Simultaneous comparison of multiple treatments: combining direct and indirect evidence. BMJ. (2005) 331:897–900. 10.1136/bmj.331.7521.897 16223826 PMC1255806

[24] Salanti G Ades AE Ioannidis JPA. Graphical methods and numerical summaries for presenting results from multiple-treatment meta-analysis: an overview and tutorial. J Clin Epidemiol. (2011) 64:163–71. 10.1016/j.jclinepi.2010.03.016 20688472

[25] Begg CB Mazumdar M. Operating characteristics of a rank correlation test for publication bias. Biometrics. (1994) 1088–101. 10.2307/2533446 7786990

[26] Egger M Smith GD Schneider M Minder C. Bias in meta-analysis detected by a simple, graphical test. BMJ. (1997) 315:629–34. 10.1136/bmj.315.7109.629 9310563 PMC2127453

[27] Brown S Hutton B Clifford T Coyle D Grima D Wells G. A Microsoft-Excel-based tool for running and critically appraising network meta-analyses–an overview and application of NetMetaXL. Syst Rev. (2014) 3:110. 10.1186/2046-4053-3-110 25267416 PMC4195340

[28] Rassy EE Bakouny Z Aoun F Haddad FG Sleilaty G Assi T. A network meta-analysis of the PD(L)-1 inhibitors in the salvage treatment of urothelial bladder cancer. Immunotherapy. (2018) 10:657–63. 10.2217/imt-2017-0190 29562804

[29] Petrylak DP de Wit R Chi KN Drakaki A Sternberg CN Nishiyama H Ramucirumab plus docetaxel versus placebo plus docetaxel in patients with locally advanced or metastatic urothelial carcinoma after platinum-based therapy (RANGE): a randomised, double-blind, phase 3 trial. Lancet. (2017) 390:2266–77. 10.1016/S0140-6736(17)32365-6 28916371

[30] Loriot Y Necchi A Park SH García-Donas J Huddart RA Burgess EF Erdafitinib (ERDA; JNJ-42756493), a pan-fibroblast growth factor receptor (FGFR) inhibitor, in patients (pts) with metastatic or unresectable urothelial carcinoma (mUC) and FGFR alterations (FGFRa): phase 2 continuous versus intermittent dosing. J Clin Oncol. (2018) 36:411 10.1200/JCO.2018.36.6_suppl.411

[31] Egger M Zellweger-Zähner T Schneider M Junker C Lengeler C Antes G. Language bias in randomised controlled trials published in English and German. Lancet. (1997) 350:326–9. 10.1016/S0140-6736(97)02419-7 9251637

