# Supplementary material for: Second-Line Systemic Treatment for Metastatic Urothelial Carcinoma: A Network Meta-Analysis of Randomized Phase III Clinical Trials
Source: Front Oncol. 2019 Jul 25;9:679. doi: 10.3389/fonc.2019.00679 (PMC6669358; doi:10.3389/fonc.2019.00679)
Supplement: Supplementary Table 1 — Ongoing clinical trials for immune checkpoint inhibitors in metastatic UCa. [file Table_1.doc]

**Supplementary Table 1.** Ongoing clinical trials for immune checkpoint inhibitors in metastatic UCa

| Clinical trial number | Drug (s) investigated | Clinical setting | Study design | Phase | Primary endpoint |
| --- | --- | --- | --- | --- | --- |
| NCT02437370 | Pembrolizumab + docetaxel or gemcitabine | Second- or third-line | Non-randomized, double arm  Arm A: pembrolizumab + docetaxel  Arm B: pembrolizumab + gemcitabine | I | MTD**^b^** |
| NCT01524991 | Ipilimumab + gemcitabine + cisplatin | Fist-line | Single-arm UC cohort | II | One-year OS^c^ |
| NCT02807636  (IMvigor130) | Atezolizumab  ± gemcitabine/ carboplatin or cisplatin | First-line | Randomized, double-blind, placebo-controlled, three-arm  Arm 1: atezolizumab + gemcitabine/carboplatin or cisplatin  Arm 2: placebo + gemcitabine/carboplatin or cisplatin  Arm 3: atezolizumab alone | III | PFS^d^, OS, AEs^e^ |
| NCT02853305  (KEYNOTE-361) | Pembrolizumab  ± gemcitabine/ carboplatin or cisplatin | First-line | Randomized, controlled, three arm  Arm 1: pembrolizumab  Arm 2: pembrolizumab + gemcitabine/carboplatin or cisplatin  Arm 3: chemotherapy (gemcitabine/carboplatin or cisplatin) | III | PFS, OS |
| NCT03036098  (CheckMate-901) | Nivolumab + ipilimumab or gemcitabine/cisplatin or carboplatin | First-line | Randomized  Experimental group: nivolumab + ipilimumab or nivolumab + gemcitabine/cisplatin or carboplatin  Comparator group  : chemotherapy (gemcitabine/cisplatin or carboplatin alone) | III | PFS, OS |
| NCT02546661 | Durvalumab  ± AZD4547 (FGFR tyrosine kinase inhibitor) | Second- or third-line | Randomized to durvalumab alone or the combination | I | AEs |
| NCT02443324 | Pembrolizumab + ramucirumab (anti -VEGFR2 antibody) | Second-line and  beyond | Non-randomized, single-arm for multiple tumors including UC | I | DLTs^f^ |
| NCT01928394  (CheckMate-032) | Nivolumab  ± ipilimumab | Any line | Randomized, single arm for multiple tumors including UC | I/II | ORR^g^ |
| NCT02516241  (DANUBE) | Durvalumab  ± tremelimumab | First-line | Three-arm randomization to durvalumab alone, durvalumab + tremelimumab, or chemotherapy (gemcitabine/cisplatin) | III | OS |
| NCT02608268 | MBG453 (TIM-3 antagonist) + PDR001 (anti-PD-1 antibody) | Second-line and  beyond | Non-randomized to MBG453 alone or MBG453 in combination PDR001 for multiple tumors including UC | I/II | AEs, ORR, DLTs |
| NCT01968109 | Nivolumab ± BMS-980616 (anti-LAG3 antibody) | Any line | Randomized, multiple tumor types including UC  Arm 1: BMS-980616 alone  Arm 2: BMS-980616 + nivolumab | I/II | AEs, ORR, DCR^h^, DOR^i^ |
| NCT02528357 | Pembrolizumab  ± GSK3174998 (OX40 agonist) | Any line | Non-randomized, multiple tumor types including UC  Part 1: GSK3174998 alone  Part 2: GSK3174998 + pembrolizumab | I | AEs, DLTs |
| NCT02178722 | Pembrolizumab  + epacadostat  (indoleamine 2,3-dioxygenase inhibitor.) | Second line and  beyond | Single-arm, multiple tumor types including UC | I/II | DLTs, ORR |
| NCT02318277 | Durvalumab  + epacadostat | Second line and  beyond | Single-arm, multiple tumor types including UC | I/II | DLTs, AEs, ORR |
| NCT02655822 | Atezolizumab  ± CPI-444 (adenosine-A2A receptor antagonist) | Any line | Randomized, multiple tumor types including UC, dose selection study of CPI-444, 1 arm combining with atezolizumab | I | DLTs, ORR, AEs, MDL^j^ |
| NCT02426125  (RANGE trial) | Docetaxel ± ramucirumab (anti -VEGFR2 antibody) | Second line and  beyond | Randomized, double-blind, placebo-controlled  Arm 1: docetaxel + ramucirumab  Arm 2: docetaxel + placebo | III | PFS |
| NCT02365597 | Erdafitinib (pan-FGFR tyrosine kinase inhibitor | Any line | Two-arm multicenter, open-Label Study | II | ORR |

^a^UC, urothelial carcinoma; ^b^MTD, maximum tolerated dose; ^c^OS, overall survival; ^d^PFS, progression-free survival; ^e^AEs, adverse events; ^f^DLTs, dose-limiting toxicities; ^g^ORR, objective response rate; ^h^DCR, disease control rate; ^i^DOR, duration of response; ^j^MDL, maximum dose level
