# Supplementary figures and images for: Second-Line Systemic Treatment for Metastatic Urothelial Carcinoma: A Network Meta-Analysis of Randomized Phase III Clinical Trials
Source: Front Oncol. 2019 Jul 25;9:679. doi: 10.3389/fonc.2019.00679 (PMC6669358; doi:10.3389/fonc.2019.00679)

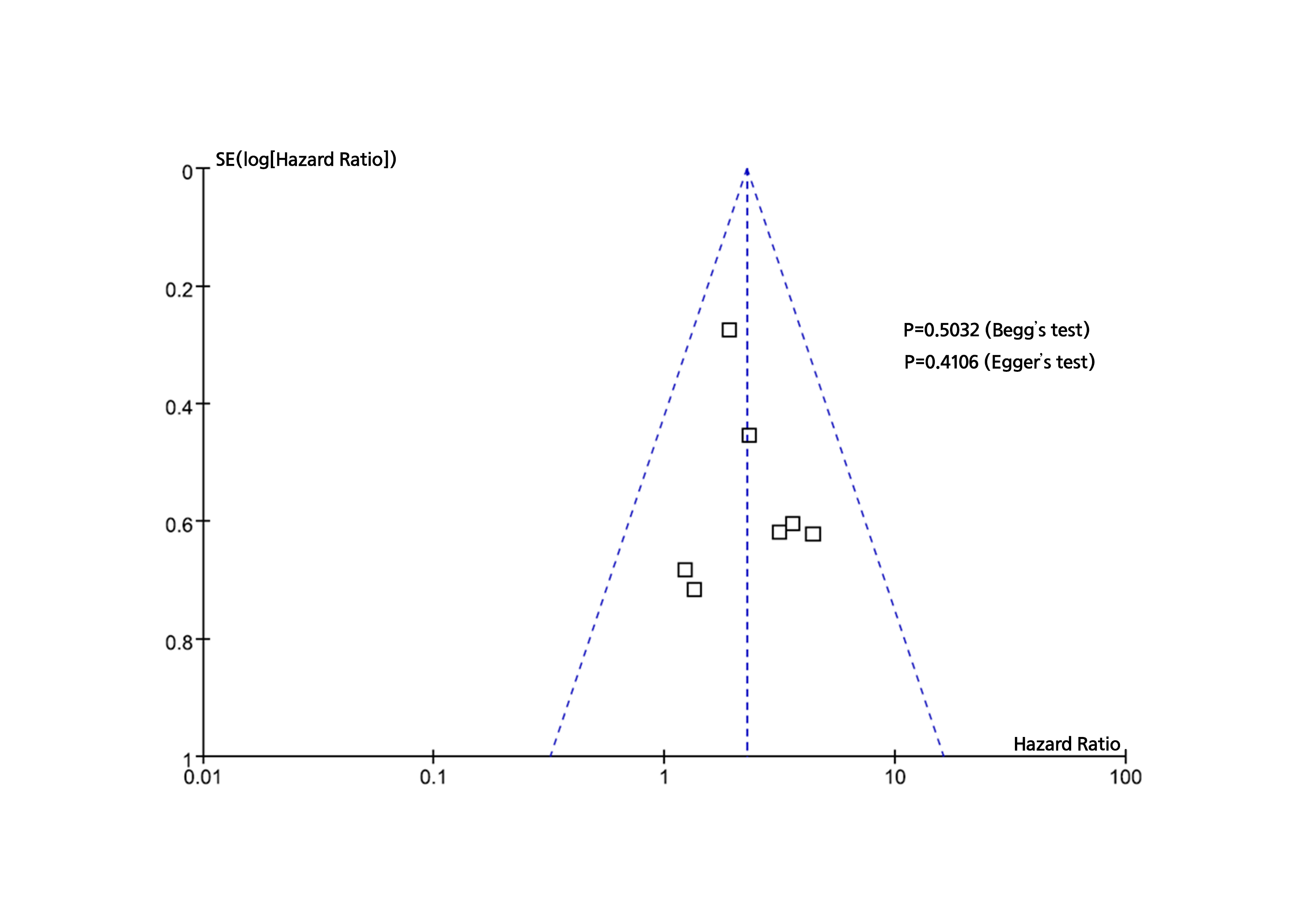

Supplement: Supplementary Figure 1 — Funnel plot for the assessment of potential publication bias in enrolled studies. Each point means a separate study for the indicated association. Vertical line indicates the mean effects size. [file Image_1.TIF]
